# 712. Dynamics of Colonization with Multi-Drug Resistant Organisms and Clinical Outcomes in Critically Ill Patients: A Prospective Cohort Study

**DOI:** 10.1093/ofid/ofad500.774

**Published:** 2023-11-27

**Authors:** Max W Adelman, Kirsten B Rydell, Andrea M Detranaltes, Rachel Atterstrom, Muhammad H Virk, Marissa G Schettino, Abigail A Amaya, Husna Malikzad, Mary N Jones, Maya Pimentel, Blake M Hanson, Samuel A Shelburne, Tor Savidge, Cesar A Arias

**Affiliations:** Houston Methodist Hospital, Houston, Texas; Houston Methodist Hospital, Houston, Texas; Houston Methodist Hospital, Houston, Texas; Houston Methodist Hospital, Houston, Texas; Houston Methodist Hospital, Houston, Texas; Houston Methodist Hospital, Houston, Texas; Houston Methodist Hospital, Houston, Texas; Houston Methodist Hospital, Houston, Texas; Houston Methodist Hospital, Houston, Texas; Texas A&M University School of Medicine, Houston, Texas; The University of Texas Health Science Center, Houston, Texas; MD Anderson-University of Texas, Houston,, Texas; Baylor College of Medicine, Houston, Texas; Houston Methodist and Weill Cornell Medical College, Houston, TX

## Abstract

**Background:**

Critically ill patients have frequent intestinal colonization with multi-drug resistant organisms (MDRO). However, the duration of colonization and its impact on clinical outcomes are unknown.

**Methods:**

We performed a prospective cohort study of adult patients admitted to ICUs at a tertiary care hospital. All patients had stool samples collected twice weekly for up to four weeks or until discharge from the ICU. Stool samples were cultured with selective media for vancomycin-resistant enterococci (VRE), extended-spectrum β-lactamase-producing Enterobacterales (ESBL-E), and carbapenem-resistant Enterobacterales (CRE). We characterized patient demographics and compared outcomes between patients with and without colonization.

**Results:**

A total of 102 patients were included. Mean age was 62 years (SD±15), 62 (61%) were men, 67 (66%) were white, and 34 (33%) were admitted from a separate healthcare facility. Thirty-nine (38%) were colonized with ≥1 MDRO at ≥1 time point. There were no statistically significant differences in baseline characteristics between patients with and without colonization. Of the 39 patients with colonization, 23 (59%) were colonized with VRE at ≥1 time point, 18 (46%) with ESBL-E, and 7 (18%) with CRE; 8 (20%) were colonized with >1 organism (**Figure 1**). Notably, most patients (13/23 with VRE, 15/18 with ESBL-E, and 4/7 with CRE colonization) were colonized with each MDRO type at only one time point during their ICU stay (**Figure 1**). In-hospital mortality did not differ according to colonization status (21% with any colonization vs. 17% without, p=0.70). When assessing mortality according to specific MDRO type, VRE colonization was associated with in-hospital mortality (35% with VRE colonization vs. 14% without, p=0.02).

Figure 1.

**Figure:**
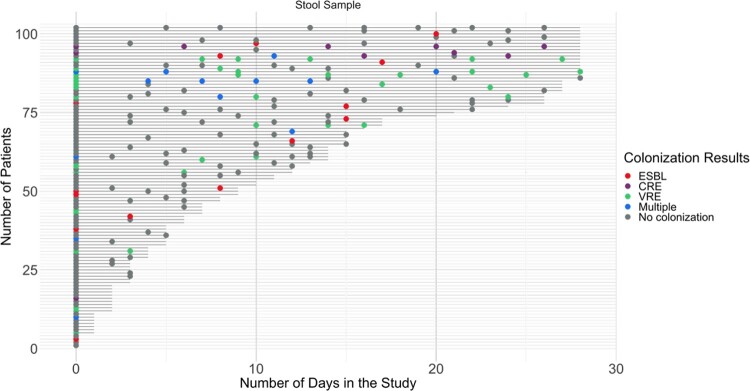

Results of serial stool samples tested for target multi-drug resistant organisms in intensive care unit patients. Each horizontal line represents a different patient included in the study (N=102) and each circle represents a separate stool sample.

**Conclusion:**

Nearly 40% of ICU patients were colonized with VRE, ESBL-E, and/or CRE, although colonization duration was generally limited, possibly reflecting antibiotic pressures and microbiome dynamics in critically ill patients. More research is needed to identify clinical, microbiome, and organism genomic factors that facilitate persistence and loss of colonization.

**Disclosures:**

**All Authors**: No reported disclosures

